# Impacts of Tooth Loss on OHRQoL in an Adult Population in Cape Town, South Africa

**DOI:** 10.3390/ijerph18094989

**Published:** 2021-05-08

**Authors:** Faheema Kimmie-Dhansay, Carla Cruvinel Pontes, Usuf M. E. Chikte, Albert Chinhenzva, Rajiv T. Erasmus, Andre Pascal Kengne, Tandi E. Matsha

**Affiliations:** 1 Division of Research and Postgraduate Studies, Faculty of Dentistry, University of the Western Cape, Cape Town 7505, South Africa; 2 Division of Health Systems and Public Health, Department of Global Health, Stellenbosch University, Cape Town 7505, South Africa; pontescarla@hotmail.com (C.C.P.); umec@sun.ac.za (U.M.E.C.); albertc1980@gmail.com (A.C.); andre.kengne@mrc.ac.za (A.P.K.); 3 Division of Clinical Pathology, Stellenbosch University, Cape Town 7505, South Africa; rte@sun.ac.za; 4 Non-Communicable Diseases Research Unit, South African Medical Research Council, Cape Town 7505, South Africa; 5 SAMRC/CPUT/Cardiometabolic Health Research Unit, Department of Biomedical Sciences, Cape Peninsula University of Technology, Cape Town 7535, South Africa; matshat@cput.ac.za

**Keywords:** quality of life, tooth loss, adults, self-concept, noncommunicable diseases

## Abstract

(1) Background: Tooth loss is an important component of the global burden of oral disease, greatly reducing the quality of life of those affected. Tooth loss can also affect diet and subsequent incidences of lifestyle diseases, such as hypertension and metabolic syndromes. This study aimed to evaluate the oral health-related quality of life (OHRQoL) score using the oral impacts on daily performance (OIDP) index in relation to tooth loss patterns among adults. (2) Methods: From 2014 to 2016, a cross-sectional study was conducted on adults living in Bellville South, Cape Town, South Africa. The OHRQoL measure was used to evaluate the impact of tooth loss. (3) Results: A total of 1615 participants were included, and 143 (8.85%) had at least one impact (OIDP > 0). Males were less likely to experience at least one impact compared to the females, OR=0.6, 95% C.I.: 0.385 to 0.942, *p* = 0.026. Those participants who did not seek dental help due to financial constraints were 6.54 (4.49 to 9.54) times more likely to experience at least one impact, *p* < 0.001. (4) Conclusions: Tooth loss did not impact the OHRQoL of these subjects. There was no difference in the reported odds for participants experiencing at least one oral impact with the loss of their four anterior teeth, the loss of their posterior occlusal pairs, or the loss of their other teeth.

## 1. Introduction

Tooth loss has been studied for decades and there are many different causes attributed to it, such as dental decay, periodontal disease, and trauma [1]. Dental caries and periodontal disease are known to be related to social and behavioural factors [1] and diet [2]. The lack of access to dental care [3], tobacco use [1], and poor oral health knowledge and oral hygiene practices [4] also play a crucial role in the progression of these oral conditions.

There are local and systemic sequelae to tooth loss [5,6]. Local complications of tooth loss can result in aesthetic and functional complications [7,8]. Aesthetic complications can affect speech [9] and lead to negative psychological consequences [10]. In addition, the loss of any teeth in the mouth can lead to malocclusion. Malaligned teeth can be difficult in terms of adequate plaque control, thus harbouring bacteria that can result in dental caries and periodontitis [11]. In addition, the loss of anterior teeth can negatively influence the ability to find work and can have an impact on daily living [12]. Missing teeth leads to the resorption of the alveolar bone, leading to ill-fitting dentures, which can also cause a poorer oral health-related quality of life (OHRQoL) [13]. The loss of teeth can also result in debilitating tempero-mandibular joint (TMJ) disorders [14]. Tooth loss has been implicated in a poorer OHRQol for different populations across the globe [15,16].

Systemic complications of tooth loss include gastroesophageal reflux disease [17] and an increased risk for diabetes mellitus [18]. The management of tooth loss includes both fixed and removable options. Dental implants are not readily available to the larger South African population due to their high cost. For this reason, a significant proportion of edentulous or partially edentulous people living in this country wear removal dentures. Many have their dentures constructed by unqualified technicians at a fraction of what it may cost if constructed by qualified oral health professionals. The consequences are often ill-fitting dentures that are prone to unwanted complications, such as localised trauma and denture stomatitis. The best way to avoid these complications is to prevent tooth loss. 

South Africa is a country known internationally for its deep history of apartheid, which has, to date, resulted in one of the highest rates of inequality in the world [19]. This has translated into major inequalities in healthcare and a lack of access to healthcare for the majority of the country’s population. Although there have been a few studies evaluating the OHRQoL in South Africa, this is the first study undertaken to evaluate the impact that tooth loss has on the OHRQoL [20,21]. The aim of this study is to assess the impact of tooth loss on the OHRQoL in an adult population in Cape Town, South Africa.

## 2. Materials and Methods

### 2.1. Study Design and Participants

This cross-sectional study was conducted from 2014 to 2016 as part of the Cape Town Vascular and Metabolic Health (VHM) study, which has been previously described in detail [22]. The Ethics Committee approved the study of the Faculty of Health and Wellness Sciences of the Cape Peninsula University of Technology (N14/01/003a). All the procedures performed in the studies involving human participants followed the institutional and/or national research committee’s ethical standards and the 1964 Helsinki declaration and its later amendments or comparable ethical standards. 

Informed consent was obtained from all the individual participants included in the study. A non-probabilistic sampling technique was employed in which adults who lived in Bellville South, Cape Town, South Africa and met the inclusion criteria were included in the study. Adults over the age of 18 years, nonpregnant women, people with no previous history of diabetes mellitus, and participants who had the ability to give informed consent to engage in the study and who had the willingness to join in the study met the inclusion criteria. Individuals who required prophylactic antibiotics, participants undergoing renal dialysis, and participants with cancer were excluded. A multistage random sampling technique was employed and previously described [23]. The sample size was based on the prevalence of 33% of difficulty in the eating domain of the oral impacts on daily performance (OIDP) tool in a previous study [20], and was estimated to be 339. South Africa is classified as an upper-middle-income country, with the highest level of inequality globally, having a Gini coefficient of 0.63 [24]. This study is compliant with the Strengthening the Reporting of Observational Studies in Epidemiology (STROBE) report [25].

### 2.2. Clinical Examination and OIPD Tool

A trained and calibrated dental examiner assessed the decayed, missing, and filled tooth (DMFT) score, and the OIDP score was recorded by trained examiners. The OIDP tool was offered to the participants in English or Afrikaans, as the community was bilingual. 

The OIPD was evaluated across ten domains: eating, speaking, cleaning teeth, light activities, vigorous activities, sleeping, relaxing, smiling, emotional state, and social contact, and the time frame was the previous six months. The OIDP score was calculated using the product of the frequency of the impact and the severity of the impact. The frequency of the impact was recorded as a 5-part Likert-type scale of every or nearly every day; once or twice a week; 3–4 times a week; once or twice a month and less than once a month. The severity of the impacts was reported as a 4-part Likert-type scale of no effect, mild effect, moderate effect, and severe effect. The OIDP domain score was calculated as the product of the severity of the impact and the frequency of each domain’s impact. The overall OIDP score was the sum of the OIDP domain scores across the ten domains divided by the maximum domain score (20) multiplied by 100 [26]. The OIDP category was dichotomised as the OIDP score being above 1 and was classified as having at least one dental impact. 

Posterior occluding pairs (POPs) were defined as the number of pairs of premolar and molar teeth occluding with each other (whether the maxillary premolars and molars are occluding with their maxillary partners). The POPs was further dichotomised into groups of less than four occluding pairs and groups of greater than or equal to four occluding pairs for the analysis. 

The number of functional teeth was described according to the number of teeth missing. Having less than ten teeth missing was categorised as a functional dentition, having between 10 and 20 missing teeth was classified as moderate tooth loss, and having between 20 and 27 missing teeth was regarded as severe tooth loss. Being fully edentulous was considered when at least 28 teeth were missing, as reported in Stock et al. [27]. The missing contribution was characterised by having at least one missing tooth. In Cape Town, South Africa, there is a tendency for this study’s population to extract their four maxillary anterior teeth. We have thus classified anterior tooth loss prevalence as a participant having all four maxillary anterior teeth extracted. 

Denture status was classified as having a denture or not having a denture. The brushing frequency was determined as brushing teeth or dentures/gums once a day, twice a day, and more than twice a day. The DMFT score was defined using the WHO’s Oral Basic Surveys Method 5th edition [28]. 

The participants’ self-reported health statuses were classified as bad, poor, fair, good, or excellent. The participants were also asked about whether they sought dental care or not due to a lack of funds.

### 2.3. Statistical Considerations

The nominal, dichotomous, and ordinal variables were initially displayed as percentages and frequencies, with group comparisons based on the chi-square test. An unadjusted and adjusted logistic regression was run to determine the associations between the variables. All the missing data were excluded. A *p*-value < 0.05 was used to characterise statistically significant results. All data were analysed using StataCorp. 2017 (Stata Statistical Software: Release 15. StataCorp LLC: College Station, TX, USA).

## 3. Results

**Figure 1:**
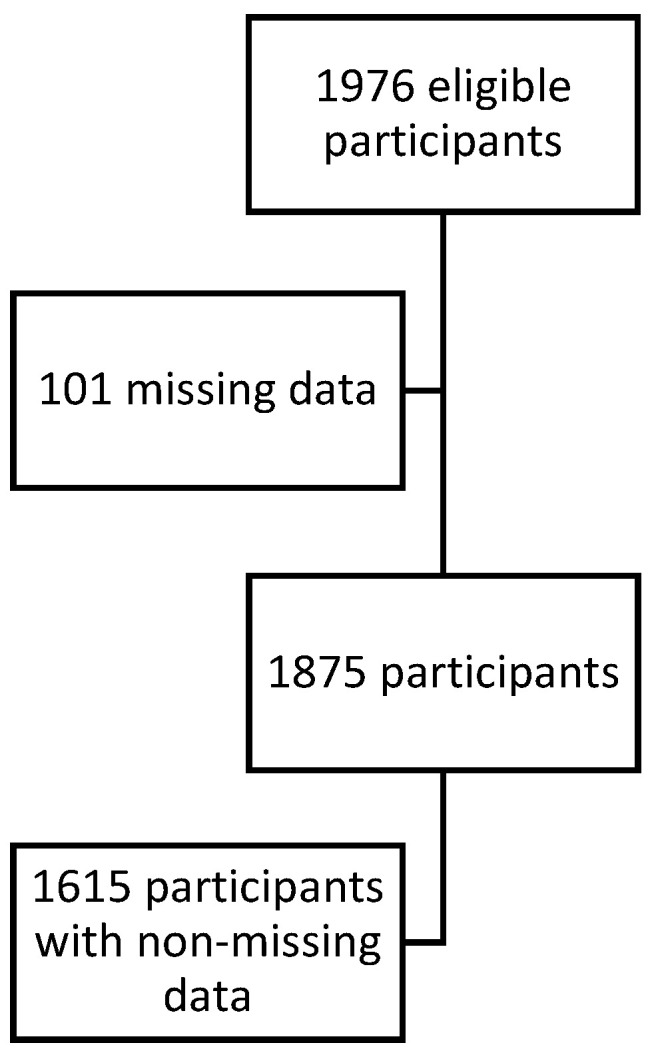
A flowchart of the number of participants.

A total of 1976 participants was initially included in the study, of which 101 were excluded due to missing data (Figure 1). The final analytic sample of 1615 participants included 408 males (25.3%) and 1207 females (74.7%). The median age was 52 (39 to 61, Table 1). There was no statistically significant difference between the gender and the age categories (*p* = 0.663). The median number of total teeth and missing teeth in the nonedentulous patients was 20 (14 to 24) and 8 (4 to 14), respectively. The median number of posterior occlusal pairs was four (one to four, Table 1).

In total, 735 participants (45.4%) of the study population had no natural teeth. Just over a third of the participants (35%; *n* = 580) had a functional dentition. Approximately 50% (*n* = 822) of the population brushed their teeth/dentures at least once a day and 124 (7.9%) reported brushing their teeth/dentures more than two times a day (Table 2). More than 40% (*n* = 661) had at least one set of posterior occlusal pairs, and of the dentate individuals with at least one missing tooth, just over 82% (*n* = 663) had no dentures (Table 2). For dentate individuals, 32.3% (*n* = 274) had all four maxillary anterior teeth missing (Table 3). 

The corrected item correlation ranged from 0.24 to 0.92, which is above the minimum recommended level of 0.20 to include items in the scale and which meets the stringent criterion of item convergent validity of >0.40. The highest Cronbach’s alpha of the scale was 0.96, with alpha values implying an excellent internal consistency.

A total of 143 (8.85%) individuals reported at least one impact. There was no difference in the OHRQoL for patients of different ages, nor for patients’ number of teeth present, number of missing teeth, number of posterior occlusal pairs, or number of lost maxillary anterior teeth (Table 3). There was no difference in the type of dentition status (functional versus edentulous status), nor the presence of dentures (Table 3) in relation to the OHRQoL. 

## 4. Discussion

The aim of this study is to assess the impact of tooth loss on the OHRQoL in an adult population in Cape Town, South Africa.

To our knowledge, this is the first study conducted in adults in South Africa and Africa that also reports the impact of tooth loss on the OHRQoL. In the present study, 8.85% of the participants reported having had at least one impact in the previous 6 months.

There was no difference in the oral health-related quality of oral life for subjects with and without the loss of the anterior teeth, with and without the loss of the posterior occlusal pairs, or with different numbers of missing teeth. According to Tan et al. [29], the presence of dental disease and poor health does not always imply a poorer quality of life, and the adaptive capacity and personal characteristics of the individual need to be considered. In a meta-analysis on the effect of tooth loss in the OHRQoL, Gerritsen et al. [15] found a significant correlation between tooth loss, a lower number of occluding pairs, and the loss of the anterior occlusal pairs and impaired OHRQoL, and the authors highlighted that the location and the distribution of lost teeth influenced the severity of the impairment. The current results are not in agreement with the results from this meta-analysis, considering that there is no difference in the odds of having at least one impact in participants who had lost all their anterior teeth. There was no difference in the oral impacts for the participants who had no posterior occlusal pairs compared to the participants with more than one occlusal pair present. The population of Cape Town has a high prevalence of dental disease and tooth loss [24,30]. The loss of teeth, especially of the anterior teeth, was not linked to a poorer oral health-related quality of life in this population. 

In this study, there was no difference in the OHRQoL for those who had a functional dentition or those who were fully edentulous, even though several studies in the literature support a lower OHRQoL in relation to increased partial tooth loss [31,32] (Table 3. While there is no simple explanation for this finding, it can be speculated that having teeth affected by oral disease leads to discomfort in daily life or that the presence of a denture is seen to be far more pleasing than the presence of dental pain. Previous studies from our group show a high degree of unmet dental treatment needs in the studied population; as such, potential inflammation, loose teeth, pain, and general discomfort have probably influenced the OHRQoL in the studied population [30,33].

Another factor to be considered is the potential perception of dental extraction as the ultimate solution for oral disease in this population sample. Our sample represents an underprivileged community that has limited access to healthcare. In addition, the practice of the extraction of healthy maxillary incisors is highly accepted from a young age [34,35]. Thus, the studied sample may have a different perception of oral health, whereby having teeth extracted is normalised and considered a permanent and beneficial solution for oral problems. Partially dentate subjects have probably experienced pain, gingival bleeding, and tooth mobility, which would have negatively impacted their OHRQoL. This correlates with the high level of edentulousness in the Western Cape province, as people have accepted this norm across generations, and edentulous subjects seem to be generally more satisfied than those who have teeth that cause discomfort [36]. 

The studied sample has limited access to oral health preventative services. The subjects not seeking dental care displayed six times higher odds of experiencing a dental impact than those seeking dental care. Hence, it is conceivable that the absence of regular dental visits leads to symptomatic rather than preventive treatment. Those with moderate tooth loss may have experienced repeated pain or may have traumatic experiences while waiting for treatment for long periods at free dental facilities. Thus, having teeth extracted might be a relief, leading to a higher perceived OHRQoL score. In a study by Ayo-Yusuf et al. [37], oral pain affected 19.4% of an adult South African sample. In low socioeconomic areas, nearly 21% of those who experienced pain over the previous 6 months did not do anything to relieve it, suggesting a limited access to oral care.

In this community, oral health has a low value, as evidenced by the low OIDP odds of disease and the high prevalence of disease and tooth loss presented in previous studies. 

## 5. Limitations

The presence of more females than males in the study could have resulted in women having a better OHRQoL than men since the study was conducted during the week, and most women in the study area are unemployed [38]. This tool was not tested for intrarater reliability for this study.

## 6. Conclusions

Within the limitations of this study, it can be concluded that tooth loss does not have an impact on the OHRQoL of these subjects. There was no difference in the reported odds of experiencing at least one oral impact for participants with the loss of their four anterior teeth, the loss of their posterior occlusal pairs, or with various teeth present in their mouths. The presence or absence of dentures did not cause a difference in the odds of reporting at least one oral impact. Women experienced higher odds of experiencing an oral impact compared to the male participants. The participants who had no self-perceived oral health satisfaction had higher odds of experiencing an oral impact than the participants with excellent, good, fair, and poor self-reported oral health. Those subjects who did not seek help due to a lack of funds had very high odds of experiencing at least one impact.

## Figures and Tables

**Table 1:** Baseline Demographics.

Variable	*n* (%)	Median (Interquartile Range (IQR))
Gender (*n* = 1615)		
Female	1207 (74.7)	
Male	408 (25.3)	
Age		52 (39 to 61)
Total no. of teeth present		20 (14 to 24)
No. of missing teeth		8 (4 to 14)
No. of posterior occlusal pairs		4 (1 to 4)
No. of maxillary anterior teeth lost		4 (0 to 4)

**Table 2:** The distribution of OIDP according to various clinical outcomes.

Variable	*n* (%)	OIDP% > 0	*p*-Value	
Gender				
Male		408 (25.3)	25 (6.1)	
Female		1207 (74.7)	118 (9.8)	0.025 *
Dentition status				
Edentulous		733 (45.4)	53 (7.2)	0.124
Severe tooth loss		56 (3.5)	8 (14.3)	
Moderate tooth loss		246 (15.2)	23 (9.4)	
Functional dentition		580 (35.9)	59 (10.2)	
Number of posterior occlusal pairs				
0		954 (59.1)	76 (7.9)	0.131
≥1		661 (40.9)	67 (10.1)	
Dentures (for NE subjects (MT > 0 and MT < 28) (*n* = 807)				
No denture		663 (82.2)	69 (10.4)	0.622
Any type of denture		144 (17.8)	17 (11.8)	
Brush				
Once a day		822 (50.9)	59 (7.2)	<0.001 *
Two times a day		669 (41.4)	59 (8.8)	
More than two times a day		124 (7.7)	25 (20.2)	
Missing contribution (for NE subjects (DMFT < 28)) (*n* = 873)				
Missing = 0		229 (26.2)	20 (8.7)	0.361
Missing ≥ 1		644 (73.7)	70 (10.9)	
Loss Ant teeth (for NE subjects (DMFT > 0 and DMFT < 28)) (*n* = 848)				
Ant Loss = 0		574 (67.7)	59 (10.28)	0.647
Ant loss = 4 teeth		274 (32.3)	31 (11.3)	
Satisfaction with oral health (self-perceived) (*n* = 873)				
Unsatisfied		441 (27.3)	79 (17.9)	< 0.001 *
Fair		678 (41.9)	38 (5.6)	
Satisfied		496 (30.7)	26 (5.2)	
Did not seek dental care due to lack of funds (*n* = 1615)				
No		1418 (87.7)	85 (5.9)	< 0.001 *
Yes		197 (12.2)	58 (29.4)	

* statistically significant; OIDP—oral impacts on daily performance; NE—non-edentulous; POP—posterior occlusal pairs; MT—Missing tooth; Ant—Anterior; DMFT—decayed, missing, and filled tooth.

**Table 3:** The logistic regression for OIDP scores with clinical parameters (OR with 95% confidence intervals).

Logistic Regression between OIDP Score > 1 and Clinical Parameters		Unadjusted Odds Ratio (OR )(95% Confidence Interval)	*p*-Value	Adjusted OR	*p*-Value
**Age**		0.99 (0.986 to 1.00)	0.747		
Total number of teeth present		0.97 (0.944 to 1.00)	0.118		
Total number of missing teeth		1.03 (0.994 to 1.06)	0.118		
Number of posterior occlusal pairs		0.93 (0.863 to 1.00)	0.085		
Number of lost maxillary anterior teeth		1.03 (0.917 to 1.16)	0.607		
**Gender**	Male	0.60 (0.385 to 0.942)	0.026 *	0.61 (0.379 to 0.975)	0.039 *
	Female	1		1	
**Dentition status**	Edentulous	1			
	Severe tooth loss	2.14 (0.962 to 4.754)	0.062		
	Moderate tooth loss	1.32 (0.793 to 2.209)	0.284		
	Functional dentition	1.45 (0.986 to 2.142)	0.059		
**Number of POP pp**	0	1			
	≥1	0.94 (0.570 to 1.551)	0.808		
**Dentures**	No denture	1			
	Any type of denture	1.15 (0.655 to 2.026)	0.622		
**Brush**	Once a day	1		1	
	Two times a day	1.42 (0.878 to 2.285)	0.154	1.39 (0.927 to 2.074)	0.111
	More than two times a day	3.37 (1.713 to 6.613)	< 0.001 *	3.09 (1.765 to 5.429)	< 0.001 *
**Loss of maxillary anterior teeth pp (DMFT ≤ 28)**	Ant Loss = 0	1			
	Ant loss ≥ 1	1.11 (0.702 to 1.765)	0.647		
**Satisfaction with oral health (self-perceived)**	Unsatisfied	3.94 (2.481 to 6.273)	< 0.001 *	3.94 (2.419 to 6.429)	< 0.001 *
	Fair	1.07 (0.642 to 1.792)	0.787	1.38 (0.805 to 2.349)	0.243
	Satisfied	1		1	
**Did not seek dental care due to lack of funds**	No	1		1	
	Yes	6.54 (4.490 to 9.538)	< 0.001 *	5.59 (3.768 to 8.321)	< 0.001 *

POP—posterior occlusal pairs; pp—per person; *- statistically significant
